# Human T-lymphotropic virus 1aA circulation and risk factors for sexually transmitted infections in an Amazon geographic area with lowest human development index (Marajó Island, Northern Brazil)

**DOI:** 10.1186/s12879-017-2859-x

**Published:** 2017-12-08

**Authors:** Samantha Assis de Aguiar, Samires Avelino de Souza França, Barbara Brasil Santana, Mike Barbosa Santos, Felipe Bonfim Freitas, Glenda Ferreira, Izaura Cayres-Vallinoto, Marluísa O. G. Ishak, Ricardo Ishak, Antonio Carlos Rosário Vallinoto

**Affiliations:** Federal University of Pará, Institute of Biological Sciences, Laboratory of Virology, Professor José da Silveira Netto Campus, Rua Augusto Correa s/no., Guama, Belém, Pará 66075-110 Brazil

**Keywords:** HTLV-1aA, Epidemiology, Marajó Island

## Abstract

**Background:**

This cross-sectional study evaluated the prevalence of infection with human T-lymphotropic virus 1 and 2 (HTLV-1 and HTLV-2) in a population from the municipalities of Anajás, Chaves, São Sebastião da Boa Vista (SSBV) and Portel in the Marajó Archipelago and correlated these data with the epidemiological characteristics of the study population.

**Methods:**

A total of 1899 biological samples were evaluated. The samples were screened for the presence of anti-HTLV antibodies using an enzyme-linked immunosorbent assay (ELISA), and infection was confirmed using conventional polymerase chain reaction (PCR), real-time PCR and nucleotide sequencing.

**Results:**

Eleven samples (0.58%) were seropositive for HTLV, but molecular analysis confirmed positivity in only two samples (0.11%). Nucleotide sequencing and phylogenetic analysis indicated that the two samples positive for HTLV-1 that were isolated in Chaves belonged to the Cosmopolitan subtype 1 (HTLV-1a) and Transcontinental subgroup (A).

**Conclusion:**

Our results confirmed the presence of Cosmopolitan Transcontinental HTLV-1 in the Marajó Archipelago, Amazon region, and the majority of the population revealed a lack of knowledge about sexually transmitted infections, which increases the risk of dissemination of HTLV and other agents.

## Background

Human T- lymphotropic virus 1 and 2 (HTLV-1 and HTLV-2) belong to the family *Retroviridae*, subfamily *Oncovirinae*, genus *Deltaretrovirus* [1]. HTLV-1 has a preferential tropism for CD4^+^ T cells, and HTLV-2 has a preferential tropism for CD8^+^ T cells [2]. HTLV-1 was initially described as the etiological agent of adult T-cell leukemia/lymphoma (ATLL) [3] and, subsequently, as the agent of a chronic neurodegenerative disease known as tropical spastic paraparesis/myelopathy associated with HTLV-1 (TSP/HAM) [4].

In 1982, HTLV infection was identified in a patient with hairy cell leukemia [5]. The virus had similarity with the virus isolated in 1980 [3]; however, some immunological markers suggested the existence of a new viral type, which was designated as HTLV-2. This viral type was rarely associated with symptoms of neurological and leukemic diseases [2], but without confirmed etiologic diagnosis.

Epidemiological studies in high-prevalence regions have shown that the modes of transmission of HTLV-1 and HTLV-2 are similar [6]. Therefore, the main routes of transmission of these viruses include blood transfusion, the sharing of contaminated syringes and needles, sexual contact and breastfeeding [7, 8].

HTLV-1 is endemic in southwestern Japan [9], parts of Africa (Benin [10], Cameroon [11] and Guinea-Bissau [12]), the Caribbean islands and South America [7, 13]. Infection with HTLV-2 is endemic in many Native American populations and intravenous drug users throughout the world [14, 15]. The Brazilian state of Pará is considered endemic for HTLV because this virus has been detected in several indigenous communities [7, 16] and the metropolitan area of Belém [16, 17].

The fight against endemic diseases is a constant challenge in Pará, particularly in the Marajó Archipelago, because primary health care is deficient. The social condition of the population of Marajó is characterized by underdevelopment, poverty, low education and limited infrastructure, which reflect the low human development index (HDI) [18].

In 2002, our research group described, for the first time, the tropical spastic paraparesis/HTLV-1 associated myelopathy (TSP/HAM) among three African-decent males residing in Santa Cruz do Arari county, Marajó Island [19]. Four years later we described the cases of HTLV in Quilombola communities in the Marajó Archipelago and associated the presence of the virus with the ethnic origin of the population [20]. Considering the low HDI observed in the archipelago and our recent report of the occurrence of HIV-1 associated with behavioral exposure factors [21], this study sought to expand the understanding of the epidemiology of infection with HTLV in the Marajó region.

## Methods

### Study population

A total of 1899 individuals (men, women and children) participated in this study, by spontaneous demand (it was enrolled all the individuals that spontaneously looked for health care at the hospitals and health centers of the villages and that agreed in take part of the study), and were distributed as follows: Anajás (*N* = 357), Chaves (*N* = 377), São Sebastião da Boa Vista (SSBV) (*N* = 373) and Portel (*N* = 792). The participants were provided an informed consent form containing information on the research objectives, methods, risks and benefits. Afterward, they answered an epidemiological questionnaire containing personal, socioeconomic and behavioral questions. Individuals younger than 18 years of age participated in the study after permission of their parents or guardians was obtained. The sample size calculation used the estimation for proportions (unilateral) with a test power of 0.90 and alpha of 0.01, considered a lower prevalence (1%) estimate in regarding to the city of Belém, capital of Pará State, using the BioEstat software version 5.3 [22].

### Sample collection

The samples were collected in hospitals and health centers that provided care in the urban and rural areas of the municipalities evaluated. Approximately 10 mL of peripheral blood was collected from each participant. Blood samples were obtained by venipuncture using a vacuum collection system and transferred to test tubes containing ethylenediaminetetraacetic acid (EDTA) as the anticoagulant. The samples were centrifuged at 3000 rpm for 10 min to obtain the plasma and cellular fractions, which were stored at −20 °C and transferred to the Laboratory of Virology of the Institute of Biological Sciences of the Federal University of Pará (Instituto de Ciências Biológicas da Universidade Federal do Pará–ICB/UFPA).

### Ethical aspects

This study was approved by the Research Ethics Committee of the Center for Hemotherapy and Hematology of Pará (Centro de Hemoterapia e Hematologia do Pará–HEMOPA) under protocol no. 0003.0.324.000–10.

### Serology

The plasma samples were analyzed for the presence of anti-HTLV-1 and anti-HTLV-2 antibodies using an enzyme-linked immunosorbent assay – ELISA (Anti-HTLV I/II SYM Solution kit, Symbiosis Diagnóstica Ltd., Brazil). The positive and indeterminate samples were subjected to molecular analysis to confirm infection and define the viral types and subtypes. Indeterminate samples were defined by OD values = 10% ≤ cut-off [1.000], following the manufacturer recommendations.

### DNA extraction

DNA was extracted from peripheral blood leukocytes using the Biopur extraction kit (Biometrix Diagnóstica, Brazil) following the manufacturer’s instructions. DNA was quantified using a Qubit 2.0 Fluorometer (Invitrogen, Life Technologies, USA). The samples considered valid for polymerase chain reaction (PCR) contained at least 20 ng of DNA. The DNA was re-extracted from the stored blood aliquot, in samples with less than 20 ng of DNA.

### Polymerase chain reaction

The pX region was amplified to confirm infection by the presence of proviral HTLV DNA in individuals who met the serological criteria [23]. The amplification reaction contained 26.25 μL of H_2_O, 4.0 μL of extracted DNA, 10.0 μL of 1.25 mM dNTP, 1.5 μL of each primer (20 pmol), 1.5 μL of 50 mM MgCl_2_, 5.0 μL of 10× buffer (50 mM KCl, 10 mM Tris-HCl, pH 8.3) and 0.25 μL of Taq DNA polymerase (5 U/μL) in a final volume of 50 μL. A second reaction step (nested PCR) was conducted using 4.0 μL of the previous amplification product under the same conditions described.

The amplification product (159 bp) was subjected to restriction fragment length polymorphism (RFLP) analysis to assess the presence of a restriction site (T/CGA) for the Taq I enzyme, which is found only in HTLV-2, to distinguish the viral types present. The RFLP analysis of the pX gene product was conducted by mixing 20.0 μL of the amplification product (nested PCR), 14.6 μL of H_2_O, 4.0 μL of buffer E (Promega, Madison, WI, USA), 0.4 μL of BSA and 1.0 μL of the restriction enzyme *Taq* I (10 U/μL, Promega, Madison, WI, USA) and incubating the reaction at 65 °C for 5 hours. The presence of the T/CGA site generated three fragments (85, 53 and 21 bp), which are present in HTLV-2 but absent in HTLV-1.

Nucleotide sequencing involved the amplification of the 5′ LTR region (788 bp) [24]. Each reaction contained 29.2 μL of H_2_O, 2.0 μL of extracted DNA, 8.0 μL of 1.25 mM dNTP, 1.5 μL of each primer (20 pmol), 2.5 μL of 50 mM MgCl_2_, 5.0 μL of 10× buffer (50 mM KCl, 10 mM Tris-HCl, pH 8.3) and 0.3 μL of Taq DNA polymerase (5 U/μL) in a final volume of 50 μL. A second reaction step (nested PCR) was conducted using 2.0 μL of the previous amplification product under the same conditions described.

The products of amplification of pX and 5′ LTR and the products of enzymatic digestion were separated by electrophoresis (100 V for 45 min) using a 3% agarose gel in 1× Tris-acetate-EDTA (TAE) buffer (40× TAE stock solution: 1.6 M Tris base, 0.8 M sodium acetate and 40 mM EDTA-Na^2^/1000 mL deionized water) and visualized under ultraviolet light using a transilluminator.

### Real time PCR

HTLV-1 infection was also confirmed by real-time PCR using TaqMan® Assays [(HTLV1F) 5′- gaacgctctaatggcattcttaaaacc-3′, (HTLV1R) 5′- gtggttgattgtccatagggctat-3′, (HTLV1 probe) 5′- FAM-acaaacccgacctaccc-NFQ-3′]. Albumin gene was used as endogenous control [Primer F) 5′-gctcaactccctattgctatcaca-3′, (Primer R) 5′-gggcatgacaggttttgcaatatta-3′, (probe) 5′- FAM-ttgtgggctgtaatcat-NFQ-3′] (Applied Biosystems, Foster City, CA, USA).

The reactions were performed in a StepOne PLUS™ Real-Time PCR System (Applied Biosystems, Foster City, CA, EUA), and for each reaction, [1X] TaqMan® Universal PCR Master Mix, [1X] of TaqMan® Assay [20X], and 20 ng DNA in a final reaction volume of 20 μL were used. After an initial incubation of 2 min at 50 °C, 10 min at 95 °C, followed 50 cycles of 50 s at 95 °C and 1 min at 60 °C.

### Nucleotide sequencing and phylogenetic analysis

The amplification products of the 5′ LTR region were purified to optimize nucleotide sequencing. Purification was performed using the PureLink® PCR Purification Kit (Invitrogen, USA). Nucleotide sequencing was based on the biochemical synthesis of DNA using the ABI PRISM™ 310 BigDye Terminator v3.1 kit Matrix Standards (Applied Biosystems) and the method of Sanger et al. [25]. The DNA strands were sequenced in both directions using the ABI PRISM 310 Genetic Analyzer (Applied Biosystems).

The genetic similarity between nucleotide sequences of the strains isolated herein and those available in the Genbank was accessed using the Basic Local Alignment Search Tool (BLAST) online platform from the National Center for Biotechnology Information (NCBI), available at https://blast.ncbi.nlm.nih.gov [26–28].

The sequence alignment of 437 bp from 5′LTR was performed using the Clustal W program implemented in BioEdit software version 7.1.9 [29]. The phylogenetic relationships among the sequence described in the present study (BRPA_146, MG321560 and BRPA_180, MG321561) and those available in the Genbank (GQ443748; BS130, EU108721; CA422, GQ443755; K344, DQ005558; HTLV06, GQ443757; K535, DQ005565; HTLV24, M37299; H5, U12804; Algerian, U12805; Pr52, U12805; OD, L76310; pyg19, JX507077, central, Y17014; Efe1, L02534; mel5) were analyzed using the MrBayes v3.2.1 program [30]. A phylogenetic tree was infered by the Bayesian method, using the HKY + I substitution model.

### Statistical analysis

The prevalence rates were estimated by calculating the number of individuals who have the desired characteristics searched at a given point in time over the total number of individuals evaluated. The values of the variables analyzed were presented as percentages.

The statistical reliability of Bayesian tree was evaluated using 1000 boostrap. The tree was viewed using the FigTree v1.4.0 program [31].

## Results

### Demographic characteristics and risk factors

In the studied population (Table 1), there was a prevalence of women in the four municipalities (72.07% in SSBV, 67.80% in Anajás, 67.81% in Portel and 73.78% in Chaves), totaling 69.81% of the 1899 individuals evaluated. The age group of 11–20 years was more prevalent in Chaves (20.15%) whereas the age group of 0–10 years was more prevalent in SSBV (27.09%), Anajás (23.99%), Portel (25.13%) and in the total population (26.04%) of all four municipalities. Most (52.01%) individuals older than 18 were married.

**Table 1:** Sociodemographic characteristics of the residents of the municipalities studied. Marajó archipelago, Pará, Brazil

Sociodemographic characteristics	SSBV		Anajás		Portel		Chaves		Total	
	n	(%)	n	(%)	n	(%)	n	(%)	n	(%)
Gender										
Female	258	(72.07)	240	(67.80)	533	(67.81)	273	(73.78)	1304	(69.81)
Male	100	(27.93)	114	(32.20)	253	(32.19)	97	(26.22)	564	(30.19)
NR*	5		3		6		7		31	
Age group										
0–10	97	(27.09)	83	(23.99)	196	(25.13)	73	(17.72)	449	(26.04)
11–20	53	(14.80)	60	(17.34)	150	(19.23)	83	(20.15)	346	(20.07)
21–30	46	(12.85)	60	(17.34)	111	(14.23)	56	(13.59)	273	(15.84)
31–40	60	(16.76)	44	(12.72)	117	(15.00)	55	(13.35)	276	(16.01)
41–50	43	(12.01)	36	(10.40)	72	(9.23)	40	(9.71)	191	(11.08)
51–60	28	(7.82)	31	(8.96)	67	(8.59)	38	(9.22)	164	(9.51)
> 61	31	(8.66)	32	(9.25)	67	(8.59)	25	(16.26)	155	(1.45)
NR*	15		11		12		7		45	
Marital status										
Married	138	(62.16)	117	(50.21)	281	(48.36)	136	(40.12)	672	(52.01)
Separated	5	(2.25)	14	(6.01)	31	(5.34)	3	(0.88)	53	(4.10)
Single	64	(28.83)	87	(37.34)	238	(40.96)	169	(49.85)	558	(43.19)
Widowed	15	(6.76)	15	(6.44)	31	(5.34)	9	(9.14)	70	(0.70)
NR* (< 18 years)	151		124		211		60		546	
Educational level										
Illiterate	33	(10.68)	50	(19.23)	153	(21.46)	37	(10.69)	273	(16.59)
Literate	190	(61.49)	132	(50.77)	397	(55.68)	186	(53.76)	905	(54.98)
Primary education	38	(12.30)	39	(15.00)	79	(11.08)	59	(17.05)	215	(13.06)
Secondary education	33	(10.68)	29	(11.05)	74	(10.38)	44	(12.72)	180	(10.94)
Higher education	15	(4.85)	10	(3.85)	28	(1.40)	20	(5.78)	73	(4.43)
NR*	64		97		61		31		253	
Family income										
< 1 minimum monthly wage	160	(52.81)	120	(44.28)	165	(22.57)	113	(33.93)	558	(34.07)
1–3 minimum monthly wage	140	(46.20)	148	(54.61)	540	(73.87)	211	(63.36)	1039	(63.43)
≥ 4 minimum monthly wage	3	(0.99)	3	(1.11)	26	(3.56)	9	(2.70)	41	(2.50)
NR*	70		86		61		44		261	

* NR: not reported

With regard to education, 905 individuals (54.98%) in the four municipalities were literate, 273 individuals (16.59%) were illiterate, 215 (13.06%) individuals had received a primary education, 180 (10.94%) had received a secondary education, and 73 (4.43%) had received a higher education. The lowest gross household incomes (less than one minimum monthly wage) were found in SSBV (52.81%). An income comprising one to three minimum monthly wages was more prevalent in Anajás (54.61%), Portel (73.87%) and Chaves (63.36%).

Table 2 shows the risk factors for the transmission of sexually transmitted infections among the study participants older than 11. Considering the four municipalities, 59.04% of the respondents did not know what sexually transmitted diseases (STDs) were. When asked about the use of condoms during sexual intercourse, 43.56% of the respondents reported that they never used them, and the municipality of SSBV presented the highest prevalence of the non-use of condoms (49.30%). When asked about their sexual partners in the four municipalities, 76.53% of the respondents reported having a steady partner.

**Table 2:** Risk factors of the population older than 11 years of age in the municipalities studied

Risk factors	SSBV		Anajás		Portel		Chaves		Total	
	n	(%)	n	(%)	n	(%)	n	(%)	n	(%)
Do you know what STDs are?										
No	127	(58.80)	142	(67.62)	287	(57.06)	146	(56.15)	702	(59.04)
Yes	89	(41.20)	68	(32.38)	216	(42.94)	114	(43.85)	487	(40.96)
NR*	60		64		93		44		261	
Do you use condoms?										
Always	43	(20.00)	53	(25.73)	112	(23.58)	59	(24.08)	267	(23.40)
Never	106	(49.30)	94	(45.63)	204	(42.95)	93	(37.96)	497	(43.56)
Sometimes	66	(30.70)	59	(28.64)	159	(33.47)	93	(37.96)	377	(33.04)
NR*	61		68		121		59		309	
Do you have a steady partner?										
Yes	162	(75.00)	162	(76.42)	367	(77.59)	186	(75.92)	877	(76.53)
No	54	(25.00)	50	(23.58)	106	(22.41)	59	(24.08)	269	(23.47)
NR*	60		62		123		59		304	
Sexual orientation										
Homosexual	3	(1.33)	18	(8.87)	23	(4.80)	5	(2.03)	49	(4.25)
Heterosexual	221	(98.22)	184	(90.64)	452	(94.36)	240	(97.56)	1097	(95.14)
Bisexual	1	(0.44)	1	(0.49)	4	(0.84)	1	(0.41)	7	(0.61)
NR*	51		71		117		58		297	
Do you use illicit drugs?										
Yes	6	(3.24)	15	(6.79)	17	(3.70)	5	(1.91)	43	(3.81)
No	179	(96.76)	206	(93.21)	443	(96.30)	257	(98.09)	1085	(96.19)
NR*	91		53		136		42		322	
Are you sexually active?										
Yes	173	(74.25)	166	(71.86)	391	(73.36)	211	(77.29)	941	(74.09)
No	60	(25.75)	65	(28.14)	142	(26.64)	62	(22.71)	329	(25.91)
NR*	43		43		63		31		180	
Age of sexual debut										
10–15 years	68	(31.63)	82	(40.59)	184	(39.48)	98	(41.18)	432	(38.54)
16–20 years	121	(56.28)	100	(49.50)	249	(53.43)	125	(52.52)	595	(53.08)
21–25 years	20	(9.30)	17	(8.42)	28	(6.01)	11	(4.62)	76	(6.78)
> 25 years	6	(2.79)	3	(1.49)	5	(1.07)	4	(1.68)	18	(1.61)
NR*	61		72		130		66		329	
Sex with sex workers										
Yes	8	(4.08)	20	(9.90)	29	(6.58)	14	(5.88)	71	(6.59)
No	188	(95.92)	182	(90.10)	412	(93.42)	224	(94.12)	1006	(93.41)
NR*	80		72		155		66		373	

* NR: not reported

With regard to sexual orientation, 95.14% of the study population claimed to be heterosexual. The municipality of Anajás presented the lowest prevalence of heterosexuals (90.64%) and the highest prevalence of homosexuals (8.87%). When inquired about the use of illicit drugs, 96.19% of the study population reported having never used them. Most (53.08%) of the respondents had initiated their sexual life at the age of 16 to 20 years. In addition, 74.09% of the study population was sexually active, and 93.41% reported not having sex with sex workers.

### Serological and molecular analyses

Of the 1899 samples tested with ELISA, 11 (0.58%) samples were positive and eight (0.42%) samples showed an indefinite pattern for antibodies against HTLV-1/2. The signal to cutoff ratio of these samples as well as data on age, sex and municipality are shown in Table 3.

**Table 3:** Serological profile for anti-HTLV-1/2 in Marajó Archipelago, Pará, Brazil

Municipality	Subjects			ELISA	
	Record	Age	Gender	Signal-to-cutoff ratio	Results
SSBV	366	91	F	1.385	Positive
SSBV	171	19	F	1.000	Indeterminate
SSBV	237	10	M	0.922	Indeterminate
SSBV	256	16	F	0.907	Indeterminate
SSBV	701	15	F	1.003	Indeterminate
SSBV	274	2	F	0.917	Indeterminate
Anajás	152	38	F	1.323	Positive
Anajás	243	37	M	1.403	Positive
Anajás	303	31	F	1.919	Positive
Anajás	117	48	F	0.905	Indeterminate
Anajás	168	14	F	0.906	Indeterminate
Anajás	169	16	F	0.948	Indeterminate
Portel	508	7	M	1.363	Positive
Portel	688	22	M	3.100	Positive
Chaves	146	53	F	2.740	Positive
Chaves	167	46	F	2.243	Positive
Chaves	180	31	F	1.399	Positive
Chaves	246	7	M	2.713	Positive
Chaves	504	18	M	3.213	Positive

*SSBV* São Sebastião da Boa Vista; Cut-off = 1.000

Of the 19 seropositive samples, only two were truly positive for HTLV infection, which was confirmed by amplification of the 159-bp fragment of the pX gene. This result indicated that the prevalence of infection with the virus in the study population was 0.11%.

These two samples showed no restriction site for the *Taq* I enzyme, which indicates infection with HTLV-1. Real-time PCR also confirmed the HTLV-1 infection in these two samples. In all of the samples, the human albumin gene was amplified. The two infected individuals (#146 and #180) lived in Chaves.

**Fig. 1:**
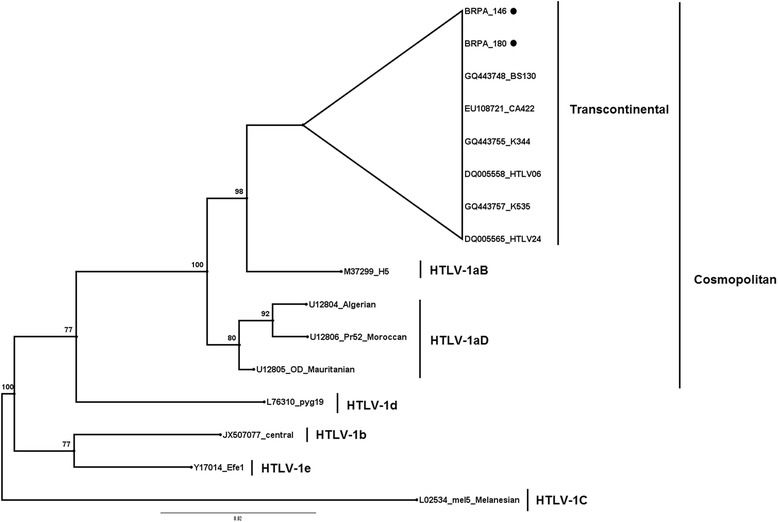
Rooted phylogenetic tree, showing the evolutionary relationship of human T-lymphotropic virus 1 strains described thus far including newly sequenced isolates from the present study BRPA_146 [MG321560] and BRPA_180 [MG321561]). The tree was constructed by the Bayesian method after alignment of 437 nucleotides of the 5’LTR region. The Mel5 isolate was used as outgroup. The statistical support was applied using 1000 bootstrap replicates. Cosmopolitan group: Transcontinental (subgroup A), Japanese (subgroup B), West African (subgroup C), North African subgroups (subgroup D). Geographical origin and ethnic origin are described in italics between parenthesis

Nucleotide sequencing and phylogenetic analysis (Fig. 1) indicated that the two HTLV-1 samples belonged to the Cosmopolitan subtype I (HTLV-1a), Transcontinental subgroup (A). One of the sequences showed an average similarity of 92.67% with 100 HTLV-1A isolates available in GenBank (Zhang et al. [27]; Morgulis et al. [28]) whereas the other sequence showed an average similarity of 89.8%.

The two infected individuals (#146 and #180) lived in Chaves. Sample #146 belonged to a woman who was 53 years old, single and homosexual and who had reported not having used illicit drugs, initiated her sexual life at the age of 10–15 years and reported never using condoms. Sample #180 belonged to a woman who was 31 years old, married and heterosexual and reported not having used illicit drugs, initiated her sexual life at the age of 16–20 years and reported never using condoms.

Table 4 shows the profile of infection with other infectious agents in the samples that were seropositive in the ELISA. Data were obtained from the serological database of the Laboratory of Virology of UFPA. The samples were positive for hepatitis A virus, hepatitis B virus, hepatitis C virus, cytomegalovirus, dengue virus, rubella virus, *Chlamydia pneumoniae*, *Chlamydia trachomatis*, *Treponema pallidum* and *Plasmodium vivax*. The antibody reactions to cytomegalovirus (100%), rubella virus (82%), hepatitis A virus (64%) and *P. vivax* (36.36%) were expressive; however, an antibody reaction to HIV was not observed.

**Table 4:** Serological profile of other infectious agents in samples positive for HTLV-1/2. Marajó Archipelago, Pará, Brazil

Sample code	366	152	243	303	508	688	146	167	180	246	504
HIV	–	–	–	–	–	–	–	–	–	–	–
HAV (IgG)	+	–	+	+	–	+	+	+	+	–	–
HBV (Anti-Bc)	–	–	–	–	–	–	–	–	+	–	–
HCV	–	–	+	–	–	–	–	–	–	–	–
CMV (IgG)	+	+	+	+	+	+	+	+	+	+	+
Dengue (IgG/IgM)	–	+	+	–	–	–	–	–	IND	–	IND
Rubella (IgG/IgM)	+	+	+	+	–	+	+	–	+	+	+
*Chlamydia pneumoniae* (IgG/IgM)	+	+	–	+	–	+	–	–	+	–	+
*Chlamydia trachomatis* (IgG/IgM)	–	+	–	+	–	–	–	–	+	–	–
*Treponema pallidum* (IgG)	IND	–	–	–	IND	–	–	–	–	–	–
*Plasmodium vivax* (IgG)	+	+	+	+	–	–	–	–	–	–	–

*HIV* human immunodeficiency virus, *HAV* hepatitis A virus, *HBV* hepatitis B virus, *HCV* hepatitis C virus, *CMV* cytomegalovirus, *IND* indeterminate result

## Discussion

The Brazilian Institute of Geography and Statistics (Intituto Brasileiro de Geografia e Estatística–IBGE) reported a small difference in the percentage of men and women distributed in the municipalities of the Marajo archipelago, with a predominance of men [32]. However, the predominance of women in our study can be attributed to the division of family labor: the men worked outside of the home, whereas the women, who worked at home, were able to attend the research stations for data collection and took their children to these visits.

There was a significant number of children and individuals younger than 20 years in the population studied. In this respect, it has been reported that the prevalence of HTLV-1 is high between the third and fifth decades of life [33]; however, in this study, the individuals in this age group accounted for only approximately 27% of the study population, and this result may have influenced the infection rate recorded in the municipalities.

The analysis of the education level indicated that the illiteracy rate was not considerable, accounting for the percentage of literate individuals and those with up to 8 years of education (primary education). However, the 16.5% of the population who were illiterate cannot be ignored because a low level of education is a risk factor for infection with HTLV [34]. These data are useful for the development of local public policies that take into consideration the characteristics and demands of the target population.

The prevalence rate of HTLV infection of 0.11% (2/1899) observed in Marajó is considered low compared to the rate of 1.8% recorded in Salvador, which is the highest rate recorded in Brazil. In some specific risk groups, including pregnant women in Mato Grosso do Sul (0.1%, 37/32,512) [35] and blood donors in Maranhão (0.15%, 561/365,564) [36], the prevalence rates were similar to those found in the municipalities of Marajo studied; however, the distinct profile of the study subjects may have influenced the results.

The occurrence of two cases of HTLV-1 in Chaves agrees with the evidence of the infection with this virus type in urban and rural populations in the Brazilian Amazon. A group of researchers in northern Brazil found a prevalence of HTLV-1 carriers of 3.06% (*N* = 259) in Quilombola communities in Marajó, where cases were found in the municipalities of Santana do Arari (2.06%) and Ponta de Pedras (1%). By contrast, a prevalence of HTLV-2 infection of 1.06% was found in Santana do Arari [20].

The prevalence of HTLV-2 has been reported among Amerindian peoples in Brazil and other countries. A high prevalence of HTLV-2 (7.8%, *N* = 1324) was found in indigenous communities in the Amazon [7], including cases in mothers and children with HTLV-2c in a closed indigenous village (Kararao–Kayapo) [16, 17]. Notably, the absence of HTLV-2 infection in the investigated populations does not exclude a possible local occurrence because the Amazon is endemic to this viral type, as previously reported.

The evidence of infection of Amerindians with HTLV-2 and infection of Quilombola communities with HTLV-1 may be associated with the phylogenetic origin of HTLV strains and their dispersion patterns across the globe. The Marajó archipelago contains indigenous populations, but the African culture was mixed in the region when the slave trade led slaves to take refuge in the archipelago in the colonial period. The contact between these communities may have contributed to the dissemination of HTLV in the Brazilian Amazon [20].

Other tests corroborate the results on the prevalence of HTLV-1 in rural communities. In Brazil, a high prevalence of HTLV-1 (Cosmopolitan Transcontinental subtype) was identified in Quilombola villages in Bahia, and a phylogenetic analysis indicated the occurrence of African migration during the slave trade [37].

Both HTLV-1 isolates from Chaves belonged to the Cosmopolitan subtype and Transcontinental subgroup (HTLV-1aA). This viral subtype is widely distributed throughout the world and is endemic in Africa, Asia and the Americas [33].

In Mozambique, the screening of blood donors and patients infected with HTLV via nucleotide sequencing of the LTR region indicated that all HTLV-1 isolates belonged to the Cosmopolitan Transcontinental subtype [38]. In Cuba, a phylogenetic study evaluated 12 symptomatic HTLV-1 carriers, and infection with this subtype was confirmed in all samples evaluated [39].

HTLV-1aA is the major circulating viral subtype in Brazil, and the states of Bahia [40], São Paulo [41] and Pará [42] have the highest prevalence rates of infection. Analyses conducted in HTLV-1 carriers in Brazil, both symptomatic (TSP/HAM) and asymptomatic, indicated that the prevalence of HTLV-1aA was greater than that of other viral subtypes and subgroups [43].

In Pará, the Cosmopolitan subtype, Transcontinental subgroup was the only subtype observed in urban and coastal areas [44], except among Japanese immigrants living in Tomé-Açu, where the Japanese subgroup was also found [45]. HTLV-1aA was described in the Brazilian Amazon with a high frequency in populations with risk of infection, including in blood donors, patients co-infected with HIV-1/HTLV-1 and Quilombola communities [20, 42, 44].

The prevalence of HTLV-1 varies according to age, gender and economic status in most areas where the virus is endemic [33]. In this context, the risk for infection is strongly associated with being female as well as having an advanced age and an unfavorable economic situation [46]. The findings of this study are consistent with those of previous studies considering that in Chaves, the cases of HTLV-1 infection were found in women older than 30, with low income. Notably, causal relationships cannot be discussed because of the low prevalence of infection within the sample universe.

The prevalence of HTLV infection increases considerably starting at age 30 and is higher among women. This result is because the retrovirus is transmitted more efficiently from men to women. The risk of spread is directly proportional to the advanced age of the male partner, high proviral load and the length of the relationship [47]. This result deserves attention because it can determine the vertical transmission of HTLV, considering that infected women tend to perpetuate the infection through childbirth or breastfeeding.

These results corroborate the prevalence of HTLV in two women in Chaves. We highlight that approximately 70% of the study sample were women, and this predominance may have influenced the results.

The main risk factors for infection with HTLV are low socioeconomic status, low education and risky sexual behavior [34]. A case-control study involving a multivariate analysis and conducted with blood donors in Bahia indicated that the risk factors for infection include self-reported history of STDs, two or more sexual partners over a lifetime and the irregular use of condoms [48].

In this study, both women reported early sexual initiation and never having used condoms. One woman had three sexual partners over her lifetime; however, both women denied having relations with sex workers or having contracted STDs. One woman did not know what STDs were. Risky sexual behavior has been shown to be a facilitator of HTLV infection [49].

With regard to the diagnostic methods, some factors may influence the occurrence of false positive results in ELISA, including a low titer of anti-HTLV antibodies, low proviral load, infection with other retroviruses and cross-reactivity against other parasitic agents, particularly those that cause malaria, which is endemic in Pará [50]. Notably, the seropositive samples were tested for the presence of other infections, including HIV and malaria, the main comorbidities producing cross-reactivity patterns in ELISA; however, none of the samples evaluated were positive for HIV infection.

Ultimately, our results suggest the low specificity of ELISA and reinforce the importance of the implementation of molecular techniques in the routine practice of health and surveillance centers to increase the reliability of epidemiological data on HTLV.

## Conclusion

Our results confirmed the presence of Cosmopolitan Transcontinental HTLV-1 in the Marajó Archipelago, Amazon region, and the majority of the population revealed a lack of knowledge about sexually transmitted infections, which increases the risk of dissemination of HTLV and other agents.

## Abbreviations

ATLL: Adult T-cell leukemia/lymphoma
BLASR: Basic Local Alignment Search Tool
EDTA: Ethylenediaminetetraacetic acid
ELISA: Enzyme-linked immunosorbent assay
HDI: Human development index
HEMOPA: Center for Hemotherapy and Hematology of Pará
HTLV: Human T-lymphotropic virus
HTLV-1/HTLV-2: Human T-lymphotropic virus 1 and 2
HTLV-1aA: Cosmopolitan subtype and Transcontinental subgroup of HTLV-1
IBGE: Brazilian Institute of Geography and Statistics
ICB/UFPA: Institute of Biological Sciences of the Federal University of Pará
NCBI: National Center for Biotechnology Information
PCR: Polymerase chain reaction
RFLP: Restriction fragment length polymorphism
SSBV: São Sebastião da Boa Vista
STDs: Sexually transmitted diseases
TAE: Tris-acetate-EDTA
TSP/HAM: Tropical spastic paraparesis/HTLV-1 associated myelopathy
